# Draft Genome Sequence of Terrestrial *Streptomyces* sp. Strain VITNK9, Isolated from Vellore, Tamil Nadu, India, Exhibiting Antagonistic Activity against Fish Pathogens

**DOI:** 10.1128/MRA.01282-20

**Published:** 2021-01-07

**Authors:** Subhasish Saha, Nabila Mohammad Ishaque, Ilia Burgsdorf, Markéta Macho, Daniela Ewe, Laura Steindler, Pavel Hrouzek, Krishnan Kannabiran, Kumar Saurav

**Affiliations:** a Laboratory of Algal Biotechnology-Centre Algatech, Institute of Microbiology of the Czech Academy of Sciences, Třeboň, Czech Republic; b Department of Biomedical Sciences, School of Biosciences and Technology, VIT University, Vellore, India; c Department of Marine Biology, Leon H. Charney School of Marine Sciences, University of Haifa, Haifa, Israel; Indiana University, Bloomington

## Abstract

We report the draft genome sequence of *Streptomyces* sp. strain VITNK9, isolated from a soil sample collected in Vellore District (12.9165°N, 79.1325°E), Tamil Nadu, India, with an assembly size of 7,920,076 bp and 72.7% GC content.

## Abstract (precis)

We report the draft genome sequence of *Streptomyces* sp. strain VITNK9, isolated from a soil sample collected in Vellore district (12.9165°N, 79.1325°E), Tamil Nadu, India, with an assembly size of 7,920,076 bp and 72.7% GC content.

## ANNOUNCEMENT

*Streptomyces* spp. have been considered one of the most prolific sources of pharmacologically active compounds for decades, contributing approximately 60% of antibiotics which are in clinical use today. To discover natural products possessing antagonistic activity against fish pathogens, we isolated a new actinomycete strain, *Streptomyces* sp. strain VITNK9, from a terrestrial soil sample from Vellore, Tamil Nadu, India, using actinomycete isolation agar (AIA) plates (1, 2). The strain had a smooth colony surface, an earthy odor, and a spiral spore formation, including aerial mycelia and substrate hyphae penetrating through agar, morphologies typical of *Streptomycetes*. The crude extract of the strain exhibited antimicrobial activity against several fish pathogens, implying that this strain can be an important resource of bioactive metabolites (1). Further, to obtain the genomic potential of VITNK9 to synthesize bioactive compounds, its genome was sequenced.

For DNA isolation, *Streptomyces* sp. strain VITNK9 was incubated on ISP1 broth at 30°C and 250 rpm for 4 days. Genomic DNA was isolated using the High Pure PCR template preparation kit (Roche) following the manufacturer’s protocol. DNA libraries were prepared using the TruSeq DNA PCR-free kit. DNA shotgun libraries were sequenced on an Illumina platform (101-bp paired-end reads) at Macrogen (South Korea), yielding a total of 6.05 Gbp of sequence with 59,886,702 reads. Sequences were trimmed using Trimmomatic version 0.36 (minlen 100, sliding window 4:20, ILLUMINACLIP:TruSeq3PE-2.fa:2:30:8) (3), and read quality was assessed using FastQC version 0.11.5 (4) prior to *de novo* assembly using SPAdes version 3.13.0 (parameters: –k 21,33,55,77,91 --careful --only-assembler --cov-cutoff auto) (5). Scaffolds of ≥2 kb were used for further analysis. Completeness and contamination were estimated with CheckM version 1.0.7 (6) based on 460 markers and using lineage workflow. For the phylogenetic analysis, sequences of the closest described type strains were obtained from EzTaxon (https://www.ezbiocloud.net/) (7). Open reading frames (ORFs) for the VITNK9 genome were identified and annotated using Rapid Annotations using Subsystems Technology (RAST) (8) with the RASTtk algorithm (https://rast.nmpdr.org/) (9).

Genome assembly of VITNK9 yielded 18 scaffolds with an *N*_50_ value of 1,271,901 bp and with the largest contig being 1,996,233 bp. The genome consists of 7,919,889 bp without ambiguous nucleotides (N) with a GC content of 72.7%, 100% estimated completeness, 0.06% contamination, and 0% heterogeneity. The VITNK9 genome contains 7,148 predicted and 73 RNA genes (68 tRNA genes). Based on EzTaxon analyses of the extracted 16S rRNA gene (876 bp) from VITNK9, the strain showed 84.11% similarity to Streptomyces rochei strain NRRL B-2410^T^.

The closest neighbors of strain VITNK9 were Streptomyces coelicolor A3(2) (score, 500), Streptomyces avermitilis MA-4680 (score, 436), Streptomyces scabiei 87.22 (score, 428), Streptomyces griseus subsp. *griseus* NBRC 13350 (score, 377), and Saccharopolyspora erythraea NRRL 2338 (score, 213) detected by RAST annotation (10). The MICs of the ethyl acetate (EA) crude extract of VITNK9 were found to be moderate against the following fish pathogens: Aeromonas hydrophila and Edwardsiella tarda (0.03 mg ml^−1^), Vibrio anguillarum and Vibrio harveyi (0.06 mg ml^−1^, and Aeromonas caviae (0.125 mg ml^−1^) (1). Furthermore, an antiSMASH version 5.1.2 analysis (11) of the VITNK9 genome revealed 30 putative biosynthetic gene clusters (BGCs). According to the prediction, 12 gene clusters possess >75% similarity to BGCs, 6 gene clusters possess 50 to 75% similarity, 8 gene clusters showed <50% similarity with known BGCs, and 4 gene clusters were detected as completely unknown. The notable BGCs found using antiSMASH prediction were in the ribosomally synthesized and posttranslationally modified peptide (RiPP) class, (lantipeptide SapB [12]), the terpene class (geosmin [13], isorenieratene [14], hopene [15], albaflavenone [16], siderophore desferrioxamine B/E [17], and ectoine [18]), nonribosmal peptide synthetase (coelibactin and coelichelin [15]), and several polyketide synthase (PKS) classes. Default parameters were used for all the tools used except where otherwise noted.

Overall, *Streptomyces* sp. strain VITNK9 possesses a high potential for diverse and bioactive secondary metabolites and can further be considered an important strain to study for bioactive compounds and their biosynthesis in the future.

### Data availability.

The genome of the *Streptomyces* sp. strain VITNK9 has been deposited at GenBank under accession number no. JACWAA000000000, assembly accession no. ASM1485402v1, BioProject no. PRJNA661553, and BioSample no. SAMN16063085. The raw reads are available under accession no. SRR12997718 in the Sequence Read Archive (SRA).

## References

[1] Ishaque Nabila M, Kannabiran K. 2018. Antagonistic activity of terrestrial Streptomyces sp. VITNK9 against Gram negative bacterial pathogens affecting the fish and shellfish in aquaculture. Rev Biol Mar Oceanogr 53:171–183. doi:10.22370/rbmo.2018.53.2.1291.

[2] Ishaque NM, Burgsdorf I, Limlingan Malit JJ, Saha S, Teta R, Ewe D, Kannabiran K, Hrouzek P, Steindler L, Costantino V, Saurav K. 2020. Isolation, genomic and metabolomic characterization of Streptomyces tendae VITAKN with quorum sensing inhibitory activity from southern India. Microorganisms 8:121. doi:10.3390/microorganisms8010121. PMC7023471 31963137

[3] Bolger AM, Lohse M, Usadel B. 2014. Trimmomatic: a flexible trimmer for Illumina sequence data. Bioinformatics 30:2114–2120. doi:10.1093/bioinformatics/btu170. 24695404 PMC4103590

[4] Wingett SW, Andrews S. 2018. FastQ Screen: a tool for multi-genome mapping and quality control. F1000Res 7:1338. doi:10.12688/f1000research.15931.2. 30254741 PMC6124377

[5] Bankevich A, Nurk S, Antipov D, Gurevich AA, Dvorkin M, Kulikov AS, Lesin VM, Nikolenko SI, Pham S, Prjibelski AD, Pyshkin AV, Sirotkin AV, Vyahhi N, Tesler G, Alekseyev MA, Pevzner PA. 2012. SPAdes: a new genome assembly algorithm and its applications to single-cell sequencing. J Comput Biol 19:455–477. doi:10.1089/cmb.2012.0021. 22506599 PMC3342519

[6] Parks DH, Imelfort M, Skennerton CT, Hugenholtz P, Tyson GW. 2015. CheckM: assessing the quality of microbial genomes recovered from isolates, single cells, and metagenomes. Genome Res 25:1043–1055. doi:10.1101/gr.186072.114. 25977477 PMC4484387

[7] Kim OS, Cho YJ, Lee K, Yoon SH, Kim M, Na H, Park SC, Jeon YS, Lee JH, Yi H, Won S, Chun J. 2012. Introducing EzTaxon-e: a prokaryotic 16S rRNA gene sequence database with phylotypes that represent uncultured species. Int J Syst Evol Microbiol 62:716–721. doi:10.1099/ijs.0.038075-0. 22140171

[8] Aziz RK, Bartels D, Best AA, DeJongh M, Disz T, Edwards RA, Formsma K, Gerdes S, Glass EM, Kubal M, Meyer F, Olsen GJ, Olson R, Osterman AL, Overbeek RA, McNeil LK, Paarmann D, Paczian T, Parrello B, Pusch GD, Reich C, Stevens R, Vassieva O, Vonstein V, Wilke A, Zagnitko O. 2008. The RAST server: Rapid Annotations using Subsystems Technology. BMC Genomics 9:75. doi:10.1186/1471-2164-9-75. 18261238 PMC2265698

[9] Brettin T, Davis JJ, Disz T, Edwards RA, Gerdes S, Olsen GJ, Olson R, Overbeek R, Parrello B, Pusch GD, Shukla M, Thomason JA, III, Stevens R, Vonstein V, Wattam AR, Xia F. 2015. RASTtk: a modular and extensible implementation of the RAST algorithm for building custom annotation pipelines and annotating batches of genomes. Sci Rep 5:8365. doi:10.1038/srep08365. 25666585 PMC4322359

[10] Overbeek R, Olson R, Pusch GD, Olsen GJ, Davis JJ, Disz T, Edwards RA, Gerdes S, Parrello B, Shukla M, Vonstein V, Wattam AR, Xia F, Stevens R. 2014. The SEED and the Rapid Annotation of microbial genomes using Subsystems Technology (RAST). Nucleic Acids Res 42:D206–D214. doi:10.1093/nar/gkt1226. 24293654 PMC3965101

[11] Blin K, Shaw S, Steinke K, Villebro R, Ziemert N, Lee SY, Medema MH, Weber T. 2019. antiSMASH 5.0: updates to the secondary metabolite genome mining pipeline. Nucleic Acids Res 47:W81–W87. doi:10.1093/nar/gkz310. 31032519 PMC6602434

[12] Kodani S, Hudson ME, Durrant MC, Buttner MJ, Nodwell JR, Willey JM. 2004. The SapB morphogen is a lantibiotic-like peptide derived from the product of the developmental gene ramS in *Streptomyces coelicolor*. Proc Natl Acad Sci U S A 101:11448–11453. doi:10.1073/pnas.0404220101. 15277670 PMC509221

[13] Jiang J, He X, Cane DE. 2007. Biosynthesis of the earthy odorant geosmin by a bifunctional *Streptomyces coelicolor* enzyme. Nat Chem Biol 3:711–715. doi:10.1038/nchembio.2007.29. 17873868 PMC3013058

[14] Iftime D, Kulik A, Hartner T, Rohrer S, Niedermeyer TH, Stegmann E, Weber T, Wohlleben W. 2016. Identification and activation of novel biosynthetic gene clusters by genome mining in the kirromycin producer *Streptomyces collinus* Tu 365. J Ind Microbiol Biotechnol 43:277–291. doi:10.1007/s10295-015-1685-7. 26433383

[15] Bentley SD, Chater KF, Cerdeno-Tarraga AM, Challis GL, Thomson NR, James KD, Harris DE, Quail MA, Kieser H, Harper D, Bateman A, Brown S, Chandra G, Chen CW, Collins M, Cronin A, Fraser A, Goble A, Hidalgo J, Hornsby T, Howarth S, Huang CH, Kieser T, Larke L, Murphy L, Oliver K, O’Neil S, Rabbinowitsch E, Rajandream MA, Rutherford K, Rutter S, Seeger K, Saunders D, Sharp S, Squares R, Squares S, Taylor K, Warren T, Wietzorrek A, Woodward J, Barrell BG, Parkhill J, Hopwood DA. 2002. Complete genome sequence of the model actinomycete *Streptomyces coelicolor* A3(2). Nature 417:141–147. doi:10.1038/417141a. 12000953

[16] Zhao B, Lin X, Lei L, Lamb DC, Kelly SL, Waterman MR, Cane DE. 2008. Biosynthesis of the sesquiterpene antibiotic albaflavenone in *Streptomyces coelicolor* A3(2). J Biol Chem 283:8183–8189. doi:10.1074/jbc.M710421200. 18234666 PMC2276382

[17] Barona-Gomez F, Wong U, Giannakopulos AE, Derrick PJ, Challis GL. 2004. Identification of a cluster of genes that directs desferrioxamine biosynthesis in *Streptomyces coelicolor* M145. J Am Chem Soc 126:16282–16283. doi:10.1021/ja045774k. 15600304

[18] Prabhu J, Schauwecker F, Grammel N, Keller U, Bernhard M. 2004. Functional expression of the ectoine hydroxylase gene (thpD) from *Streptomyces chrysomallus* in *Halomonas elongata*. Appl Environ Microbiol 70:3130–3132. doi:10.1128/aem.70.5.3130-3132.2004. 15128576 PMC404422

